# Supplementary material for: Light Deficiency Inhibits Growth by Affecting Photosynthesis Efficiency as well as JA and Ethylene Signaling in Endangered Plant Magnolia sinostellata
Source: Plants (Basel). 2021 Oct 22;10(11):2261. doi: 10.3390/plants10112261 (PMC8618083; doi:10.3390/plants10112261)
Supplement: Supplementary file 1 [file plants-10-02261-s001.zip › plants-1407709-supplementary.pdf]

**Supplementary Figures and Tables**

## 1.1 Supplementary Figures

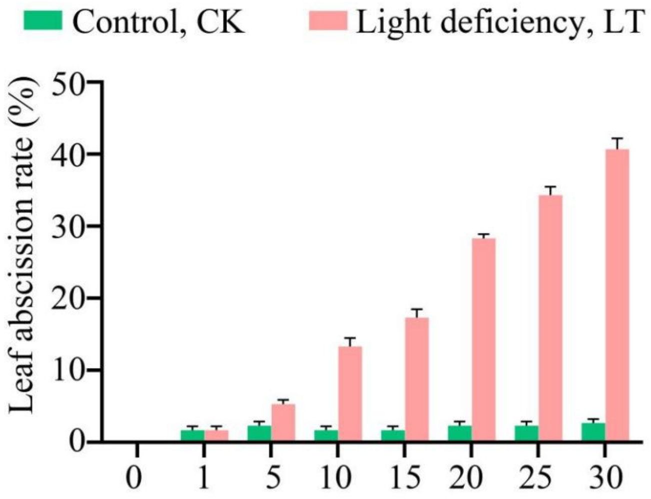


Figure S1. Changes in leaf abscission rate during shading treatment.

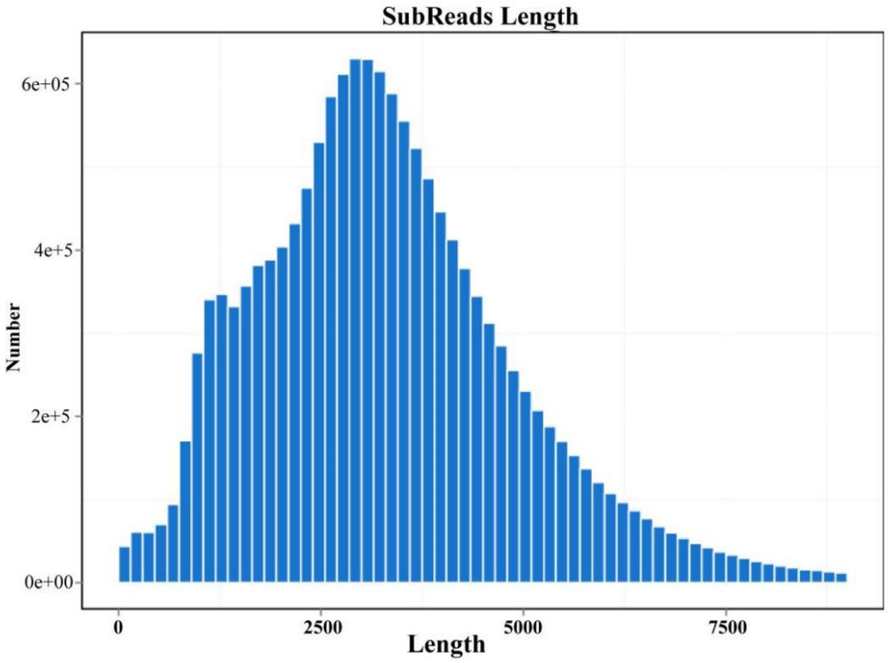


Figure S2. Subreads length distribution.

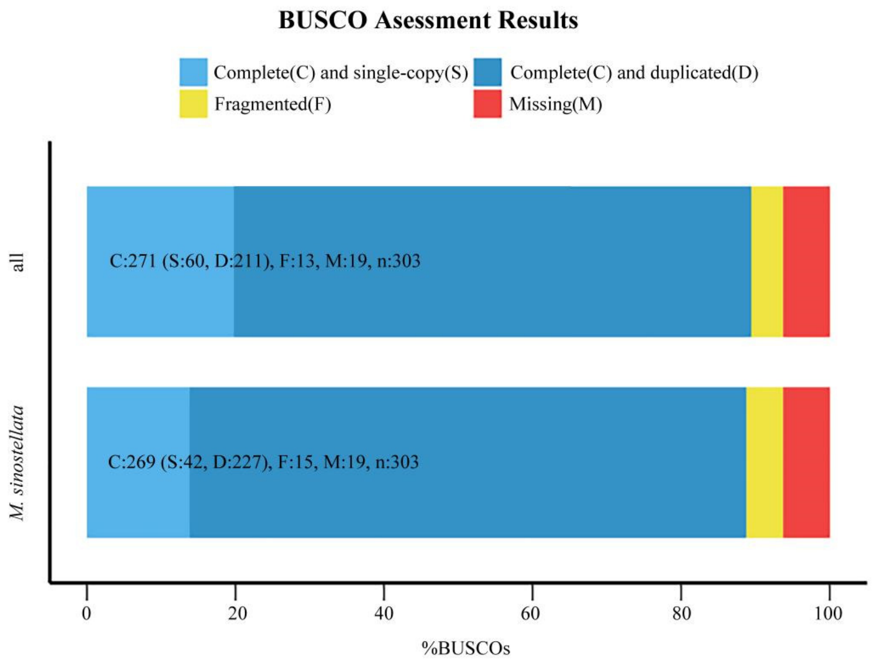


Figure S3. BUSCO assembly evaluation results.

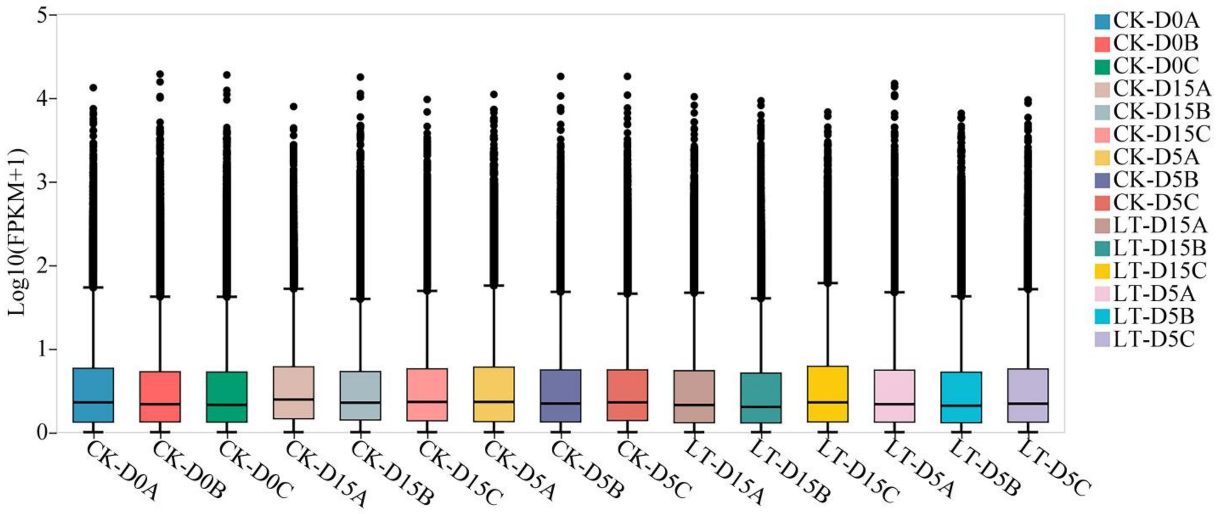


Figure S4. Box plot of expression levels 15 samples.

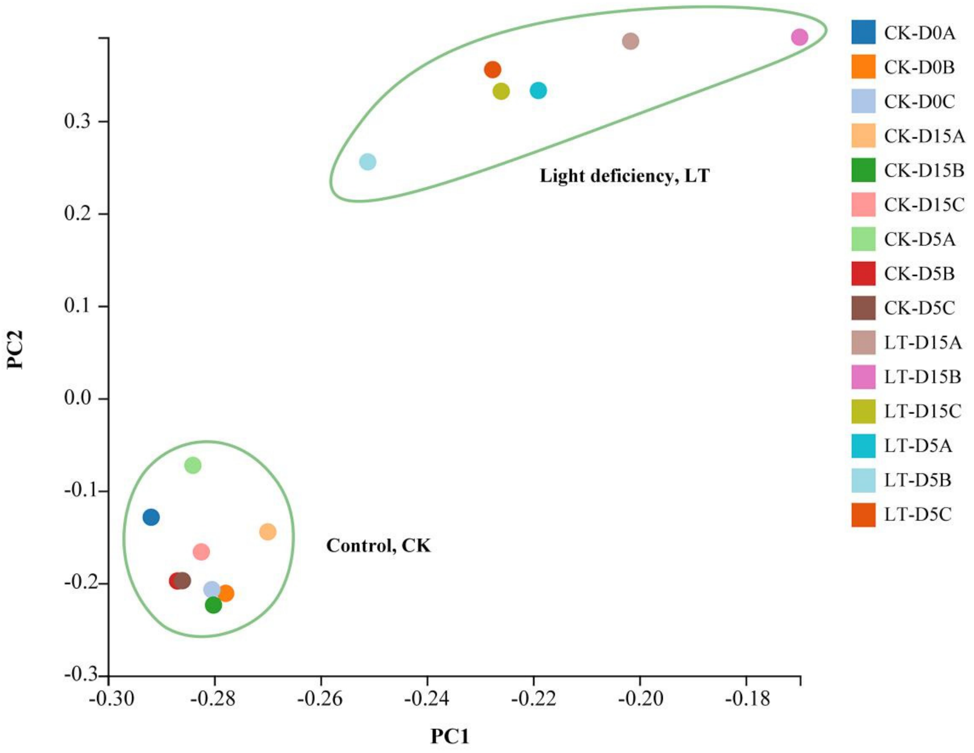


Figure S5. Principal component analysis of 15 samples.

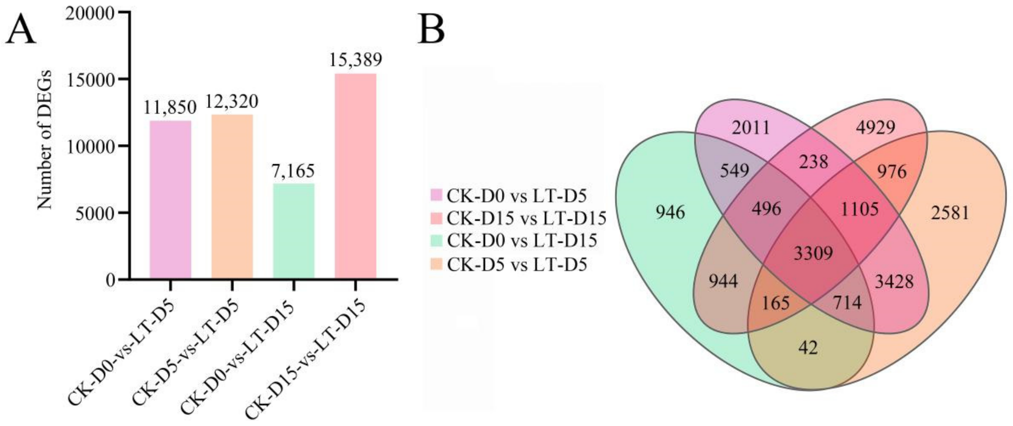


Figure S6. Light deficiency responsive DEGs identified in *M. sinostellata*. (**A**) Number of DEGs dectected in four comparison groups. (**B**) Venn dragram of DEGs commonly or specifically expressed in *M. sinostellata* under light deficiency.

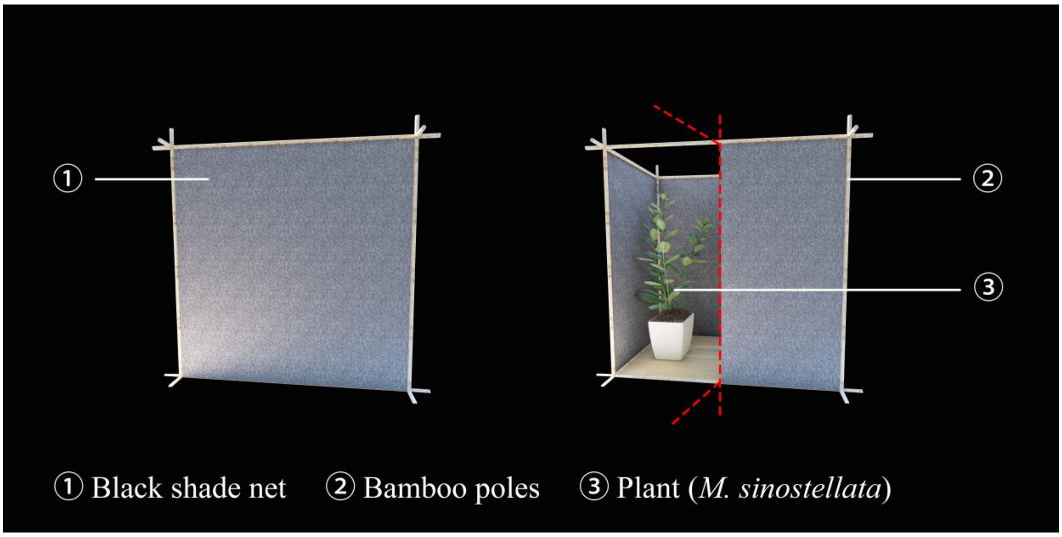


Figure S7. Light deficiency treatment device used in this research.

## 1.2 Supplementary Tables

Table S1. Primers for quantitative real time PCR (RT-qPCR)

| Gene ID | Gene name | Forward (Reverse) primer sequence | Annealing temperature Tm (℃) |
|---|---|---|---|
| isoform_10996 | EIN3 | F-GGATAAGATGACGGCGAAGG | 55.81 |
| | | R-TGGGTTGAGGTGGCGACAGA | 59.50 |
| isoform_105980 | ERF | F-GAGTCATACTACCTTCCCTGTT | 57.45 |
| | | R-CGTCTCGCTCGTGCCAATCA | 59.50 |
| isoform_14782 | JMT | F-ATGGTCCTGGCACTGTTGGG | 59.50 |
| | | R-GAGCGACTGTGCTAGGAGGC | 61.55 |
| isoform_25394 | COI1 | F-GGTATCCCTGAGGAAGTTGG | 57.45 |
| | | R-AAAGGACGATGGGTAAAGTG | 53.35 |
| isoform_39422 | JAZ | F-CTTAAATCAAGAACCGAACC | 57.45 |
| | | R-GCAATCTGGAAGGGAAAGGT | 55.40 |
| isoform_3645 | JAR1 | F-GCCAGAGCCCTTGGTTACTT | 57.45 |
| | | R-TCGTCATGGATATGCCGACT | 55.40 |
| isoform_82864 | MYC2 | F-GGCTGCAATAGAGCAATGGG | 55.00 |
| | | R-ACGAAGGAAAGCGGTGGTGA | 55.00 |

Table S2. Summary of the final transcript sequence obtained after de-redundancy.

| Total_number | Total_length | N50 | N90 | Max_length | Min_length | Sequence_GC(%) |
|---|---|---|---|---|---|---|
| 246481 | 270112156 | 1466 | 573 | 20445 | 199 | 43.97% |

Table S3. Summary of Function annotation result statistics.

| Values | Number | Percentage |
|---|---|---|
| Total | 246,481 | 100% |
| Nr | 173,103 | 70.23% |
| Nt | 146,820 | 59.57% |
| Swissprot | 128,216 | 52.02% |
| KEGG | 135,136 | 54.83% |
| KOG | 128,718 | 52.22% |
| Pfam | 107,462 | 43.60% |
| GO | 138,676 | 56.26% |
| Intersection | 58,204 | 23.61% |
| Overall | 191,343 | 77.63% |

Table S4. Summary of filtered Reads quality statistics.

| Sample | Total Raw Reads (M) | Total Clean Reads (M) | Total Clean Bases (Gb) | Clean Reads Q20 (%) | Clean Reads Q30 (%) | Clean Reads Ratio (%) |
|---|---|---|---|---|---|---|
| CK-D0A | 47.33 | 43.56 | 6.53 | 96.84 | 92.16 | 92.03 |
| CK-D0B | 45.57 | 42.24 | 6.34 | 96.99 | 92.49 | 92.68 |
| CK-D0C | 47.33 | 43.46 | 6.52 | 96.76 | 92 | 91.83 |
| CK-D5A | 47.33 | 43.11 | 6.47 | 97.36 | 93.31 | 91.09 |
| CK-D5B | 47.33 | 43.3 | 6.49 | 96.84 | 92.19 | 91.48 |
| CK-D5C | 45.57 | 42.53 | 6.38 | 96.93 | 92.37 | 93.33 |
| CK-D15A | 47.33 | 43.27 | 6.49 | 96.91 | 92.29 | 91.43 |
| CK-D15B | 45.57 | 41.95 | 6.29 | 96.62 | 91.65 | 92.04 |
| CK-D15C | 45.57 | 42.21 | 6.33 | 96.78 | 91.94 | 92.62 |
| LT-D5A | 47.33 | 43.36 | 6.5 | 96.92 | 92.34 | 91.61 |
| LT-D5B | 47.33 | 43.53 | 6.53 | 97.22 | 92.98 | 91.98 |
| LT-D5C | 45.57 | 42.06 | 6.31 | 96.85 | 92.19 | 92.3 |
| LT-D15A | 47.33 | 43.27 | 6.49 | 96.84 | 92.09 | 91.43 |
| LT-D15B | 45.57 | 41.99 | 6.3 | 96.87 | 92.19 | 92.13 |

| | | | | | | |
|---|---|---|---|---|---|---|
| LT-D15C | 45.57 | 42.27 | 6.34 | 96.81 | 92.13 | 92.75 |

Table S5. Summary of reference gene sequence alignment results.

| Sample | Total Clean Reads (M) | Total Mapping(%) | Uniquely Mapping(%) |
|---|---|---|---|
| CK-D0A | 43.56 | 86.99 | 5.03 |
| CK-D0B | 42.24 | 81.44 | 5.61 |
| CK-D0C | 43.46 | 81.74 | 5.62 |
| CK-D5A | 43.11 | 86.31 | 4.91 |
| CK-D5B | 43.3 | 82.56 | 6.15 |
| CK-D5C | 42.53 | 75.85 | 5.9 |
| CK-D15A | 43.27 | 73.76 | 7.05 |
| CK-D15B | 41.95 | 75 | 5.73 |
| CK-D15C | 42.21 | 79.81 | 6.71 |
| LT-D5A | 43.36 | 82.6 | 6.87 |
| LT-D5B | 43.53 | 81.75 | 6.72 |
| LT-D5C | 42.06 | 81.75 | 6.77 |
| LT-D15A | 43.27 | 80.36 | 7.42 |
| LT-D15B | 41.99 | 76.92 | 8.62 |
| LT-D15C | 42.27 | 80.58 | 6.72 |

Table S6. Information of TIFY gene family in *M. sinostellata.*

| Gene name | Gene ID | ORF (bp) | Protein size (aa) | Protein Molecular Weight/KD | PI | Instability index | Aliphatic index | Grand average of hydropathicity | Predicted subcellular localization |
|---|---|---|---|---|---|---|---|---|---|
| *MsTIFY3* | isoform_43991 | 522 | 173 | 18012.31 | 9.25 | 75.78 | 71.27 | -0.317 | Nucleus |
| *MsTIFY5a* | isoform_129175 | 378 | 125 | 14014.22 | 8.95 | 87.67 | 81.20 | -0.425 | Nucleus |
| *MsTIFY5b* | isoform_42079 | 366 | 121 | 13799.76 | 9.50 | 79.78 | 76.69 | -0.615 | Nucleus |
| *MsTIFY6* | isoform_18683 | 1155 | 384 | 40619.09 | 9.57 | 50.54 | 70.47 | -0.273 | Nucleus |
| *MsTIFY9* | isoform_28136 | 660 | 219 | 23991.24 | 9.73 | 45.29 | 74.89 | -0.430 | Nucleus |
| *MsTIFY10a* | isoform_29142 | 660 | 219 | 23539.77 | 9.67 | 48.11 | 59.36 | -0.594 | Nucleus |
| *MsTIFY10b* | isoform_152838 | 552 | 183 | 20696.08 | 7.64 | 58.97 | 50.66 | -0.685 | Nucleus |

Table S7. Information of mTERF gene family in *M. sinostellata.*

| Gene name | Gene ID | ORF (bp) | Protein size (aa) | Protein Molecular Weight/KD | PI | Instability index | Aliphatic index | Grand average of hydropathicity | Predicted subcellular localization |
|---|---|---|---|---|---|---|---|---|---|
| *MsmTERF1* | isoform_236198 | 936 | 311 | 35867.86 | 9.74 | 54.65 | 99.94 | -0.176 | Nucleus |
| *MsmTERF3* | isoform_139197 | 477 | 158 | 18110.01 | 9.51 | 75.66 | 80.82 | -0.166 | Nucleus |
| *MsmTERF4* | isoform_6244 | 1569 | 522 | 59592.25 | 6.42 | 43.02 | 95.9 | -0.151 | Chloroplast |
| *MsmTERF6* | isoform_96760 | 495 | 164 | 19052.56 | 9.67 | 52.86 | 99.21 | -0.164 | Chloroplast |
| *MsmTERF7* | isoform_213883 | 1215 | 404 | 45975.92 | 9.77 | 53.92 | 94.58 | -0.05 | Cell membrane/ Chloroplast |
| *MsmTERF10* | isoform_16020 | 870 | 289 | 32640.27 | 8.54 | 49.38 | 97.13 | -0.165 | Chloroplast |
| *MsmTERF12* | isoform_173710 | 660 | 219 | 24587.52 | 7.53 | 39.21 | 87.67 | -0.201 | Nucleus |

Table S8. The light intensity and quality in different conditions.

| Condition | PAR | R/FR ratio |
|---|---|---|
| Control group | 648 | 1.1 |
| Light deficiency treated group | 162 | 1.09 |
| Natural light (without shade) | 972 | 1.12 |
| Natural light (under canopy shade) | 170 | 0.043 |
